# Spin Hall photoconductance in a three-dimensional topological insulator at room temperature

**DOI:** 10.1038/s41467-017-02671-1

**Published:** 2018-01-23

**Authors:** Paul Seifert, Kristina Vaklinova, Sergey Ganichev, Klaus Kern, Marko Burghard, Alexander W. Holleitner

**Affiliations:** 1 Walter Schottky Institut and Physik-Department, Technische Universität München, Am Coulombwall 4a, D-85748 Garching, Germany; 2 Max-Planck-Institut für Festkörperforschung, Heisenbergstrasse 1, D-70569 Stuttgart, Germany; 3 Terahertz Center, University of Regensburg, D-93040 Regensburg, Germany; 4 Institut de Physique, Ecole Polytechnique Fédérale de Lausanne, CH-1015 Lausanne, Switzerland

## Abstract

Three-dimensional topological insulators are a class of Dirac materials, wherein strong spin-orbit coupling leads to two-dimensional surface states. The latter feature spin-momentum locking, i.e., each momentum vector is associated with a spin locked perpendicularly to it in the surface plane. While the principal spin generation capability of topological insulators is well established, comparatively little is known about the interaction of the spins with external stimuli like polarized light. We observe a helical, bias-dependent photoconductance at the lateral edges of topological Bi_2_Te_2_Se platelets for perpendicular incidence of light. The same edges exhibit also a finite bias-dependent Kerr angle, indicative of spin accumulation induced by a transversal spin Hall effect in the bulk states of the Bi_2_Te_2_Se platelets. A symmetry analysis shows that the helical photoconductance is distinct to common longitudinal photoconductance and photocurrent phenomena, but consistent with optically injected spins being transported in the side facets of the platelets.

## Abstract (web summary)

While the spin generation in topological insulators is well studied, little is known about the interaction of the spins with external stimuli. Here, Seifert et al. observe a helical, bias-dependent photoconductance at the lateral edges of topological Bi_2_Te_2_Se platelets for perpendicular incidence of light, distinct to common longitudinal photoconductance phenomena.

## Introduction

Selectively addressing the two-dimensional surface states (SSs) of three-dimensional (3D) topological insulators (TIs)^1–4^ in electrical transport experiments is challenging due to the intrinsic doping of TIs, resulting in sizable contribution of the bulk states (BSs) to the conductivity. Nevertheless, electrically induced spin-polarized currents due to spin-momentum locking^5^ have been demonstrated in lateral spin valve devices with a ferromagnetic spin detector based on a number of bismuth and antimony chalcogenides^6–9^. Alternatively, the SSs and their dynamics can be probed via optical excitation of photocurrents, whose decay enables access to the spin-relaxation time of both SSs and BSs in 3D Tis^10–16^. Time-resolved ARPES experiments have demonstrated dynamics control of spin-polarized currents in Sb_2_Te_3_^17,18^ and Bi_2_Se_3_^11,19^, as well as emergent Floquet-Bloch states due to hybridization of the SSs and pulsed circularly polarized light (CPL)^20^. According to theory^21,22^, CPL can couple to the electron spin in a material and the helicity of the incident photons determines the spin-polarization of the resultant in-plane photocurrents due to asymmetric excitation of spin-up and spin-down carriers. This circular photogalvanic effect has been experimentally demonstrated in Bi_2_Se_3_^23–25^, Sb_2_Te_3_^26^, and (Bi_1−*x*_Sb_*x*_)_2_Te_3_^27^ and can be tuned via electrostatic gating^24^, Fermi energy tuning^27^, and proximity interactions^28^. In addition, a helicity-dependent photovoltaic effect has been reported for normally incident light in vicinity of the metal contacts on a Bi_2_Se_3_ nanosheet^29^.

Importantly, along with the SS-generated in-plane spin polarization, the strong spin-orbit coupling (SOC) of the bulk bands can give rise to out-of-plane spin polarization diffusing perpendicularly to the applied current direction via the intrinsic spin Hall effect^30–34^. Theory further predicts that a bulk-mediated diffusion of spin density can take place between the top and bottom TI surfaces^30^. So far, only little experimental data are available on bulk spin currents in 3D TIs. Spin-charge conversion via the inverse spin Hall effect has been demonstrated for a TI/metallic ferromagnet heterostructure^35^. In general, however, SOC-enhanced scattering suggests strongly reduced spin diffusion lengths in 3D TIs, which makes spin detection and manipulation challenging.

In the present work, we explore the spin texture of Bi_2_Te_2_Se (BTS) platelets under electrical current flow using two complementary experimental methods, namely helical photoconductance measurements and magneto-optical Kerr microscopy. Our data is fully consistent with a bulk-spin Hall effect generating a spin accumulation in the side-facets of the BTS platelets, and an optical spin-injection, which can be read-out as a helical photoconductance with a lifetime characteristic of the SSs in the platelets’ side-facets.

## Results

### Helicity dependent photoconductance spectroscopy

**Fig. 1:**
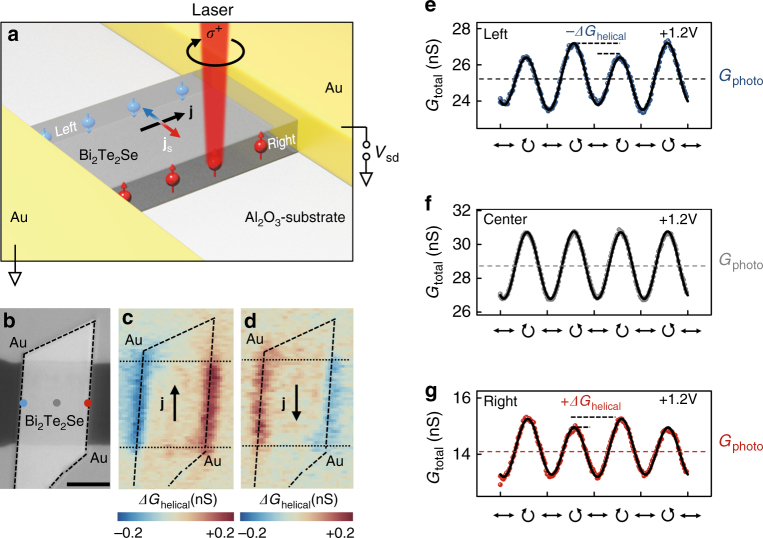
Helicity-dependent edge conductance at room temperature. **a** Sketch of a Bi_2_Te_2_Se (BTS) platelet in a metal/topological-insulator/metal geometry excited by circularly polarized light at perpendicular incidence. Red (blue) spheres represent the electron spin polarization on the right (left) facet of the platelet, when a bias voltage *V*_sd_ is applied between source to drain. Correspondingly, an electron current density **j** flows, which in turn gives rise to a transverse spin current density **j**_s_. Under illumination, the helicity of the photon excites spins, which are equally driven into this transverse direction. **b** Optical microscope image of a BTS platelet (highlighted by dashed lines), contacted by two Ti/Au contacts. Scale bar is 5 µm. **c**, **d** Spatial map of the photoconductance generated by the circularly polarized light at bias voltages of *V*_sd_ = + 1.2 V and *V*_sd_ = -1.2 V respectively. We use a photo-elastic modulator detecting the difference *ΔG*_helical_ = *G*_helical_(*σ*^−^) – *G*_helical_(*σ*^+^) of photoconductance which depends on the circularly left (*σ*^−^) and right (*σ*^+^) polarized light. **e-g** Total photoconductance *G*_total_ as a function of the laser polarization at the left and right edge, as well as the center [cf. blue, gray, and red dots in **b**]. The polarization is controlled by a quarter waveplate. The symbol **↔** denotes linearly polarized, **↻** circularly right polarized light, **↺** circularly left polarized light. All measurements are performed at *V*_sd_ = + 1.2 V, *P*_laser_ = 230 µW, and room temperature

We contact individual BTS platelets on a transparent sapphire substrate by two Ti/Au contacts, and focus a circularly polarized laser with a photon energy of 1.54 eV normally incident onto the platelets (Fig. 1a and Methods). We apply a bias voltage *V*_sd_ between the contacts, such that a local electron current density **j** flows in the sample (black arrow in Fig. 1a). Figure 1b presents an optical microscope image of an exemplary device, comprising a BTS platelet with a width of ~9 µm and a height of 90 nm, while the Au-contacts have a distance of 10 µm. We define three positions, specifically the left edge (blue circle), the center (gray circle), and the right edge (red circle). We record the photoconductance signal upon scanning the laser with a spot size of ~1.5 µm across the platelet (Fig. 1c, d), while the light polarization is modulated at a frequency much slower than any carrier-carrier or carrier-phonon interaction rates (see Methods section). The corresponding photoconductance maps acquired under an applied bias voltage *V*_sd_ of + 1.2 V (Figs. 1c) and −1.2 V (Fig. 1d) display a helicity dependent photoconductance with opposite sign at both edges. In particular, these signals reflect the difference *ΔG*_helical_ = *G*_helical_(*σ*^+^)–*G*_helical_(*σ*^−^) of the photoconductance *G*_helical_, where *σ*^+^ (*σ*^−^) corresponds to right (left) circular polarization of the incoming light. The absence of a photoconductance signal away from the edges is consistent with the in-plane spin polarization associated to the SSs at the top/bottom surface of the platelet and the normal incidence of the circularly polarized light^18^. In order to gain further insight into the origin of the edge signals, we use a quarter wave plate to resolve the sign change of *G*_helical_ as a function of helicity. Figure 1e–g show the corresponding photoconductance *G*_total_, which equals the conductance under illumination and the dark conductance subtracted via a lock-in detection (cf. Methods). The obtained helicity-dependent photoconductance difference *ΔG*_helical_, as well as the polarization-independent photoconductance *G*_photo_ are highlighted in Fig. 1e to g. We note that the main oscillation in *G*_total_ follows the linear polarization of the excitation, and it stems most likely from an anisotropy of the absorption at a finite bias under a linearly polarized excitation^36–38^ (cf. Supplementary Fig. 1 and Supplementary Fig. 2). Importantly, this linear contribution of the photoconductance does not show any significant features at the platelets’ edges (cf. Supplementary Fig. 3). In the following, we interpret *G*_helical_ to occur in the topological side surfaces of the BTS platelets as a consequence of a spin-current caused by the bulk spin-Hall effect^30–34^. In this scenario, the spin accumulation induced by the bias-driven current density **j** (black arrow in Fig. 1a, c, d) is characterized by an out-of-plane spin polarization pointing in opposite directions at the left and right edge of the BTS platelet (blue and red arrows).

### Helicity-dependent vs. longitudinal photoconductance

**Fig. 2:**
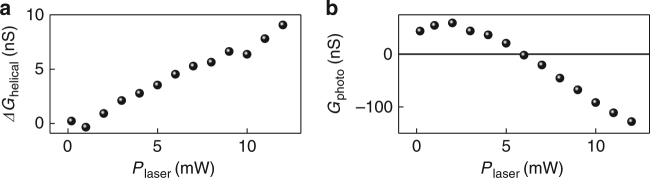
Irradiance dependence of helical and polarization-independent photoconductance. **a** The helical *ΔG*_helical_ is linear up to the largest laser powers investigated, and can be tuned to be positive or negative. The polarity depends on the excitation position and the direction of the dark current in the platelets. We show *ΔG*_helical_ for a right edge of a BTS platelet at a positive bias *V*_sd_ = + 0.55 V. **b** The polarization-independent photoconductance *G*_photo_ of the BTS platelets shows a non-linear power dependence. The non-linearity is an interplay of saturating photoconductance contributions of the surface and bulk states of the BTS platelet (saturating for *P*_laser_ ≥ 5 mW) and a negative bolometric photoconductance dominating at large laser powers [cf. ref. ^12^]. All measurements are performed at room temperature

**Fig. 3:**
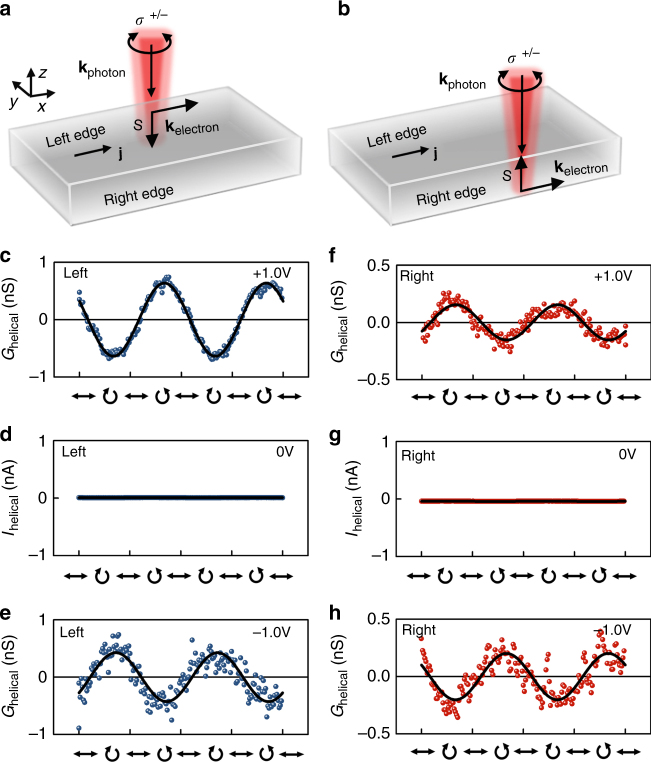
Helicity vs. bias and facet dependence of helical photoconductance. **a, b** Sketch of a BTS platelet with dark current **j** along the *x*-direction, when a bias *V*_sd_ is applied. The current defines the direction of the electron wave vector **k**_electron_ at the left and right edge. The electron spin-polarization **S** is perpendicular to **k**_electron_ and points in opposite directions at the two edges. In **a** [**b**], the left [right] edge is excited with a circularly polarized light with the photon wave vector **k**_photon_ pointing downwards. **c–e** *ΔG*_helical_ vs. the laser helicity at the left edge for *V*_sd_ = + 1 V (**c**), 0 V (**d**), and –1 V (**e**). The polarization is tuned with a quarter waveplate for an excitation scheme as sketched in **a**). **f-h** Similar data for the right edge for an excitation scheme as sketched in **b**. All measurements are performed at *P*_laser_ = 230 µW and room temperature

Figure 2a reveals that *ΔG*_helical_ depends linearly on the laser power *P*_laser_ (up to 12 mW), without reaching saturation. By contrast, the polarization-independent photoconductance *G*_photo_ displays a polarity change vs. laser power, as discussed in detail in [12] (Fig. 2b). Correspondingly, we attribute the polarity change to a saturation of the lifetime-limited photoconductance in the bulk states and the one in the SSs at the top and bottom surface for *P*_laser_ ≥ 5 mW, while a bolometric photoconductance with a negative sign dominates the high power regime. We note that a bolometric photoconductance is driven by a heated phonon bath of the BTS platelets^12^. Figure 2a highlights that *ΔG*_helical_ captures an effect that is clearly distinguished from the conventional longitudinal photoconductance effects in the bulk and surface states of BTS. This difference becomes particularly evident at a laser power of ~6 mW, where the polarization-independent *G*_photo_ is zero, while *ΔG*_helical_ can be fully controlled with the laser polarization. The latter observation indicates that *ΔG*_helical_ is not a modulation of *G*_photo_. In other words, for an absorption effect, *ΔG*_helical_ would follow *G*_photo_ in amplitude and direction. To a first approximation, *ΔG*_helical_ is decoupled from the helicity independent longitudinal photoconductance, which justifies our approach of subtracting the helicity independent conductance contributions as an offset and only evaluating the helicity dependent photoconductance (cf. Fig. 3 and Supplementary Fig. 1).

### Symmetry considerations for the helicity dependent photoconductance

The schematic sketches of the excitation configuration of our photoconductance experiment in Fig. 3a, b define the electron and photon wave vectors **k**_electron_ and **k**_photon_, as well as the spin polarization **S** at the left and right edges of the BTS platelet. As apparent from Fig. 3c to h, a finite *G*_helical_ is observed only at a finite bias voltage, thereby excluding *G*_helical_ as a photocurrent effect which would have a finite magnitude at zero bias. We note that for *V*_sd_ = 0 (Figs. 3d and 3g), the originally detected signal *I*_helical_ ~ 0 nA is plotted instead of *G*_helical_, as an experimental conductance is not defined at zero bias. As another relevant observation, *G*_helical_ flips by *π* when either the bias *V*_sd_ is reversed (cf. Fig. 3c vs. 3e and Fig. 3f vs. 3 h), or when the laser spot position is exchanged between the left and right platelet edge.

Table 1 summarizes the experimentally observed symmetry characteristics of *G*_helical_ as concluded from Figs. 1–3, and contrasts them with those of the polarization-independent *G*_photo_ and of helical photocurrents in materials with a broken symmetry^39,40^. The latter type of current is denoted as *I*_edge_ in the table, in order to associate it with the circular photogalvanic effect which is well documented for topological insulators^21–25,40^. From the observed bias dependence, any photocurrent effect can be excluded as origin of *G*_helical_. However, it should be emphasized that such currents indeed emerge at the side-facets of the BTS platelets, although their amplitude is very small (cf. Supplementary Fig. 4). The small amplitude is explainable by the fact that the optical cross section of the side facets is about 100 times smaller compared to that of the bulk volume in the BTS platelets. It is furthermore noteworthy that the helical photoconductance *G*_helical_ does not depend on the direction of the photon wavevector **k**_photon_ (cf. Fig. 3 vs. Supplementary Fig. 5 and Supplementary Fig. 6). Moreover, from the polarity change of *G*_helical_ with *V*_sd_, one can exclude a longitudinal photoconductance response based upon the following argument. In case of a longitudinal response, the sign of *G*_helical_ would reflect the preferred current direction in *x*-direction upon laser illumination. From a symmetry perspective, a current reversal is equivalent to a simultaneous operation going from left edge to right edge and a rotation of the sample by a spatial angle of *π*. The latter combined operations have to be an even operation due to rotational symmetry, while a bias reversal of *G*_helical_ is odd (cf. Table 1), which contradicts the experimental findings. Nonetheless, there remains a process which is fully consistent with the observed symmetries, specifically a transverse photoconductance. In fact, the latter can effectively modulate the conductivity along the edges of the BTS platelets, and thus supports the assumption of a bulk spin current in 3D TIs, which has been analytically predicted based upon topological arguments^30^. Recently, bulk spin currents have been identified as a possible spin generation mechanism in 3D topological insulators via the spin Hall effect^30–34^. Indeed, this mechanism would yield an accumulated spin polarization parallel to the SS-based spin polarization in the side facets of BTS platelets (cf. red and blue arrows in Fig. 1a), as recently verified and discussed^30–35^. Furthermore, it should result in an opposite spin accumulation at the opposite edges of the BTS platelets, and also reverse sign upon reversing the longitudinal charge current direction.

**Table 1:** Symmetry chart of *G*_helical_ and *G*_photon_ compared to edge photocurrent phenomena

	*G*_helical_	*G*_photo_	*I*_edge_
*V*_sd_ → *– V*_sd_	−1	−1	0
*σ*^+^ → *σ*^−^	−1	+1	−1
**k**_photon_ → *–* **k**_photon_	+1	+1	+1
left edge → right edge	−1	+1	−1

Symmetry characteristics of *ΔG*_helical_, *G*_photo_, and edge photocurrent effects *I*_edge_ for changing *V*_sd_ to −*V*_sd_, *σ*^+^ to *σ*^*,*−^, **k**_photon_ to −**k**_photon_, and the excitation position from the left to the right edge of the BTS platelets. A value −1 states a sign change under the symmetry operation, while + 1 states a sign conservation. The value 0 denotes that *I*_edge_ is independent from **j** and the bias.

**Fig. 4:**
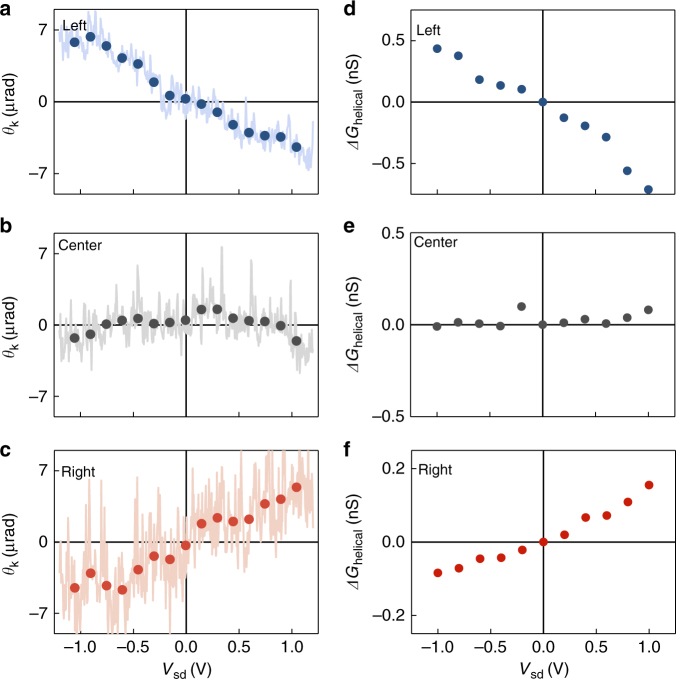
Kerr angle and helical photoconductance vs. bias voltage. **a–c** Kerr angle *θ*_k_ vs. the applied bias *V*_sd_ at the left edge (**a**), center (**b**), and right edge (**c**) of a BTS platelet (*P*_laser_ = 400 µW). **d–f** Corresponding *ΔG*_helical_ vs. the applied bias *V*_sd_ (*P*_laser_ = 230 µW). All measurements are performed at room temperature

In order to verify the presence of vertically aligned spins accumulated at the edges of the BTS platelets, we investigate the device by magneto-optical Kerr-microscopy. The detected Kerr angles *θ*_k_ (Figs. 4a–4c) are bias-dependent and their sign is opposite at the opposite edges of the platelet. Likewise, the sign of *θ*_k_ at the edges switches with the sign of the applied bias (Figs. 4a and 4c), whereas *θ*_k_ vanishes at the center of the platelet (Fig. 4b). In close correspondence, also *G*_helical_ follows such a bias and position dependence (Figs. 4d to 4f).

## Discussion

The above outlined mechanism of helicity dependent photoconductance in BTS can be rationalized by a microscopic model as follows. The spin relaxation of photogenerated charge and spin carriers in 3D TIs, including BTS, occurs on ultrafast timescales on the order of 100 fs^11^. This fast relaxation suggests that the underlying spin dynamics of *G*_helical_ are governed by spin diffusion rather than a spin precession process^31^. The spin diffusion length in Bi_2_Se_3_ was experimentally determined to be below 10 nm at room temperature^41^. This length is significantly smaller than both, the diffusion length of hot carriers that ranges between 200 and 300 nm in bismuth-chalcogenides^42^, and the laser spot size of ~1.5 µm in our experiment. Moreover, we observe that *G*_helical_ is located at the edges of the BTS platelets (Fig. 1d). Interestingly, we can observe the edge-localized *G*_helical_ also in a BTS platelet with a spatial width as small as *d*_platelet_ ~300 nm (cf. Supplementary Fig. 7). The latter observation is consistent with a dominating spin diffusion length smaller than ½ ˑ*d*_platelet_ ~ 150 nm at the edges. It has been reported that the inverse spin Hall effect in the bulk states of *n*-doped Bi_2_Se_3_ is comparable to or even dominating over the surface Edelstein effects^35,41^. Hence, a bulk spin Hall effect should be favorable over an out-of-plan spin component due to warping effects in the top and bottom surface of the BTS platelets^21^. Moreover, the side facets are expected to exhibit a spin-momentum locking^43^ even in the presence of disorder. Under sufficiently high surface disorder, the spin relaxation time has been predicted to be increased similar as the D’yakonov–Perel mechanism in a motional narrowing regime^30^. Analogously, the spin generation efficiency and surface conductivity are suggested to be strongly enhanced at the disordered facets of the BTS platelets^30^, as previously demonstrated for surfaces of BiSbTeSe_2_^44^. Along these lines, we interpret *G*_helical_ as a measure of optically excited spins which are driven by the spin Hall mechanism to (or away from) the edges (blue and red arrows in Fig. 1a), where they experience an increased (or decreased) conductivity. This scenario is able to account for all our experimental findings. In particular, it explains not only the polarization dependence in Figs. 1e to 1g, but also all other experimental observations summarized in Table 1. This interpretation suggests that *G*_helical_ is a read-out of a bulk spin current by scattering optically injected spins into the surface states of the side-facets in the BTS platetes^30^. Regarding the carriers involved in the optical excitation, it is important that we do not claim that the spin-polarization is generated in the topological surface states. Instead, we interpret the spin-polarization to predominantly originate from topologically trivial bands. In this context, it is relevant that, in particular for high doping levels, also the topologically trivial conduction band in BTS exhibits a strongly anisotropic spin-orbit splitting close to the surface^45^. The same holds for the valence bands which are energetically buried 0.8-1.6 eV below the Fermi-level^46^. Importantly, we determine the decay time of *G*_helical_ to be *τ*_decay_ = (463 ± 13) ps (cf. Supplementary Fig. 8). This value, while exceeding the spin relaxation time by far, agrees very well with the surface lifetime of the BTS platelets^12,47^; thereby strongly supporting the above mechanism. In other words, after a pulsed laser excitation, *G*_helical_ prevails as long as *τ*_decay_ within the surface states of the side facets.

Overall, the possibility to optically detect the spin Hall effect-driven spins at structurally disordered 3D TI surfaces relaxes the technical demands on device fabrications, thus underscoring the theoretical prediction that surface disorder in 3D TIs might even be beneficial for spintronic applications^30^. Furthermore, the demonstrated read-out principle might prove useful also for probing the spatial accumulation of spins in related materials such as Weyl semimetals.

## Methods

### Bi_2_Te_2_Se platelet growth and charge carrier density

BTS platelets are grown by a catalyst-free vapor-solid method on Si substrates covered by a 300 nm thick SiO_2_ layer. Bi_2_Se_3_ and Bi_2_Te_3_ crystal sources (99.999% purity) are heated up to ~580 °C in a tube furnace. Ultrapure argon gas transports the evaporated material to the growth substrates, which are heated to 450°−480 °C during a growth time of 30 min. The pressure in the furnace is maintained at 75–85 mbar at Argon flow rate of 150 sccm. Thus obtained platelets have a length in the range of 5-60 µm, a lateral width of 0.05-10 µm, and a thickness of 15–200 nm. The platelets are mechanically transferred onto Al_2_O_3_ substrates and lithographically contacted by metal strips made of thermally evaporated Ti/Au (10 nm/250 nm). Four-terminal Hall-measurements on single BTS platelets with a width of a few hundreds of nm revealed n-doping character, with an electron concentration of 10^19^ cm^−3^ at room temperature.

### Time-integrated photoconductance spectroscopy

For the excitation of the BTS platelets, we use a Ti-sapphire laser that emits light at laser pulses with 150 fs pulse duration and a repetition rate of 76 MHz. In all cases, the photon energy is set to be 1.54 eV. The position of the laser spot with size ~1.5 µm is set with a spatial resolution of ~100 nm. The helical photoconductance is measured with a standard lock-in measurement technique, where the laser is modulated by an optical chopper at a frequency of  *f*_chop_ = 2.7 kHz or a piezoelastic modulator (PEM) at a frequency of 50 kHz. The PEM is set to switch the polarization of the exciting laser between *σ*^−^ and *σ*^+^ polarized light. Alternatively, we utilize a quarter wave plate to control the circular polarization of the laser. The pulsed excitation scheme allows us to compare our time-integrated photoconductance results with our lifetime measurements from autocorrelation spectroscopy (cf. Supplementary Fig. 8) and with earlier results (see ref. [12]). We do consider our time-integrated measurements as steady state results and we can reproduce them with a CW excitation (cf. Supplementary Fig. 9). We performed the helicity resolved photoconductance experiments on three different devices. In all cases, we observed the described signals of opposite sign at the edges. All presented experiments are performed at room temperature and in a vacuum chamber at ~5 × 10^−6^ mbar.

### Autocorrelation photoconductance spectroscopy

For characterizing the decay dynamics of *G*_helical_, we split the pulsed laser beam into a pump and a probe pulse with comparable intensity, and focus the pump- (probe-) from the front (back) onto the BTS platelets (cf. Supplementary Fig. 8). The probe-beam, which is modulated by an optical chopper at a frequency of ~1930 Hz, hits the sample at a time delay *Δt*. The time-resolved autocorrelation signal is then detected with a lock-in amplifier on the sum-fequency of the pump- and the probe-pulse as a function of the time delay *Δt*. The probe-pulse is delayed via a mechanical delay stage.

### Magneto-optical Kerr microscopy

For the Kerr-effect spectroscopy we utilize the same measurement setup as for the photoconductance spectroscopy. The laser is polarized purely linearly. The polarization of the reflected light is rotated by 45° via a half-waveplate. Afterwards, a Wollaston prism splits the reflected light into two cross-polarized beams with an angle of 20° which are detected by two balanced photodetectors. The difference-signal and sum-signal of the detectors are detected by two lock-in amplifiers at a chopper-frequency of 2.7 kHz. To calibrate the reflected signal, the laser-spot is positioned onto a normally reflecting surface (e.g., gold) and the half-waveplate is adjusted, such that the difference signal of the detectors is perfectly zero. Any rotation of the reflected light’s polarization shifts the balance between the detectors, which is then detected in the difference- but not in the sum-signal. Hence the sum-signal is used to calibrate the difference-signal in order to get rid of laser-intensity based noise and fluctuations.

### Data availability

All relevant data that supports our experimental findings is available from the corresponding author upon reasonable request.

## Electronic supplementary material

Supplementary Information
