# Supplementary material for: Spin Hall photoconductance in a three-dimensional topological insulator at room temperature
Source: Nat Commun. 2018 Jan 23;9:331. doi: 10.1038/s41467-017-02671-1 (PMC5780383; doi:10.1038/s41467-017-02671-1)
Supplement: Supplementary file 1 — Supplementary Information [file 41467_2017_2671_MOESM1_ESM.pdf]

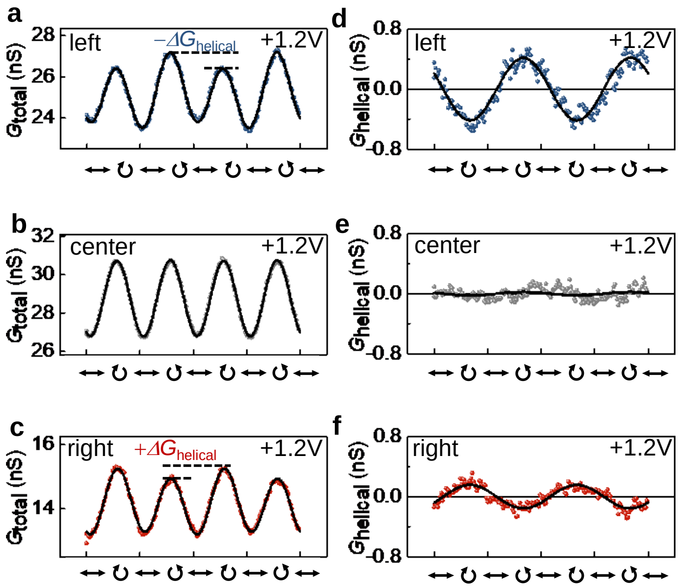

Supplementary Figure 1 | Fitting procedure of the helical photoconductance. **a, b, c,** Total photoconductance vs the polarization of the exciting photons as depicted in Figs. 1e, 1f, and 1g of the main manuscript before substracting a background which depends on the linear polarization of the photon and a background which is independent of the polarization. The helicity-dependent contribution is indicated by -$\Delta G_{\mathrm{helical}}$ and +$\Delta G_{\mathrm{helical}}$. **d, e, f,** Helical photoconductance G_{helical} which depends on the circularly right (σ^{+}) and left (σ^{-}) polarized light of (a)-(c) after substracting the background. All measurements are performed at P_{laser} = 230 µW and room temperature.

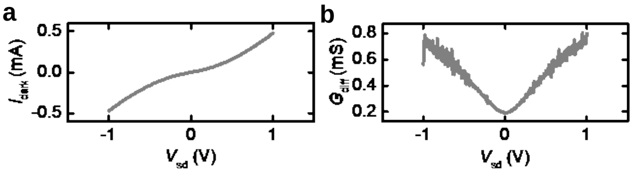

Supplementary Figure 2 | Dark current and differential conductance a, Dark current of the Bi_2Te_2Se platelet as in Figure 1 of the main manuscript vs of the applied bias voltage V_{sd} without illumination. **b** Differential (dark-) conductance G_{diff}, calculated from (**a**).

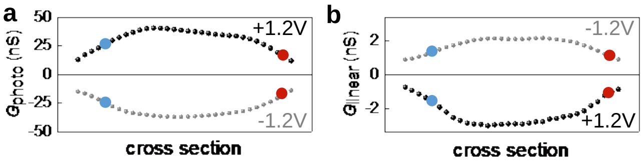

Supplementary Figure 3 | Lateral Photoconductance distribution and response to linear polarization. a, Lateral cross-section of the total photoconductance G_{total} across the BTS platelet along the blue and red points as in Figure 1 of the main manuscript for V_{sd} = +/- 1.2 V. **b,** Corresponding cross section of the photoconductance depending on the linear polarization G_{linear}. The latter is the main oscillation as in Figure 1 e-g of the main manuscript. All measurements are performed at P_{laser} = 230 µW and room temperature.

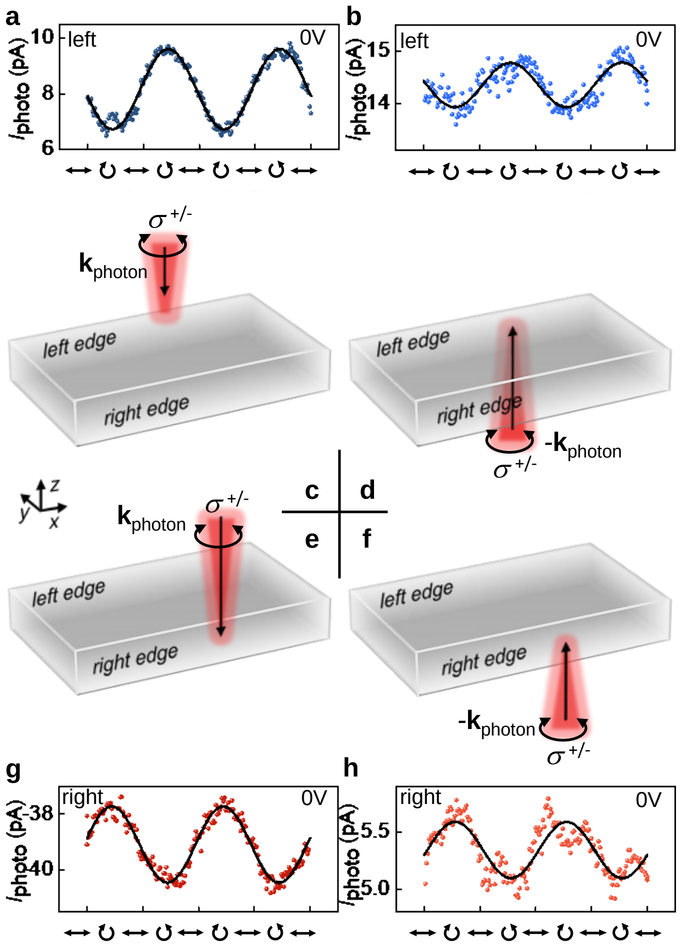

Supplementary Figure 4 | Helicity dependent edge photocurrents at zero bias. a, and **b,** Photocurrent I_{photo} at zero bias (V_{sd} = 0 V) for exciting the left side facet of the platelet for a photoexcitation incident from the top (a) and backside (b) of the platelet. The polarization is controlled with a quarter waveplate. **c,** and **d,** Schematic illustrations for an excitation at the left side facet with a $\mathbf{k}_{photon}$ pointing downwards (c) and upwards (d). **e,** and **f,** Schematic illustrations for an excitation at the right side facet with a $\mathbf{k}_{photon}$ pointing downwards (e) and upwards (f). **g,** and **h,** I_{photo} at zero bias (V_{sd} = 0 V) for exciting the right side facet of the platelet for a photoexcitation incident from the top (g) and from the backside (h). The polarization is controlled with a quarter waveplate.

Importantly, we note that for the side facets, the incidence occurs effectively at an oblique angle of ~90°. Hereby, photocurrents, such as the circular photogalvanic, are possible.[35] However, I_{photo} exhibits a rather small amplitude of only pA, which is 1000x smaller than the $G_{helical}$ at V_{sd} = 1V. The small signal of I_{photo} is consistent with the optical cross section of the side facets of only ~10^{-2} of the overall laser spot of ~1.5 µm. All measurements are performed at P_{laser} = 300 µW (photoexcitation from the top) and P_{laser} = 500 µW (photoexcitation from the bottom) at room temperature.

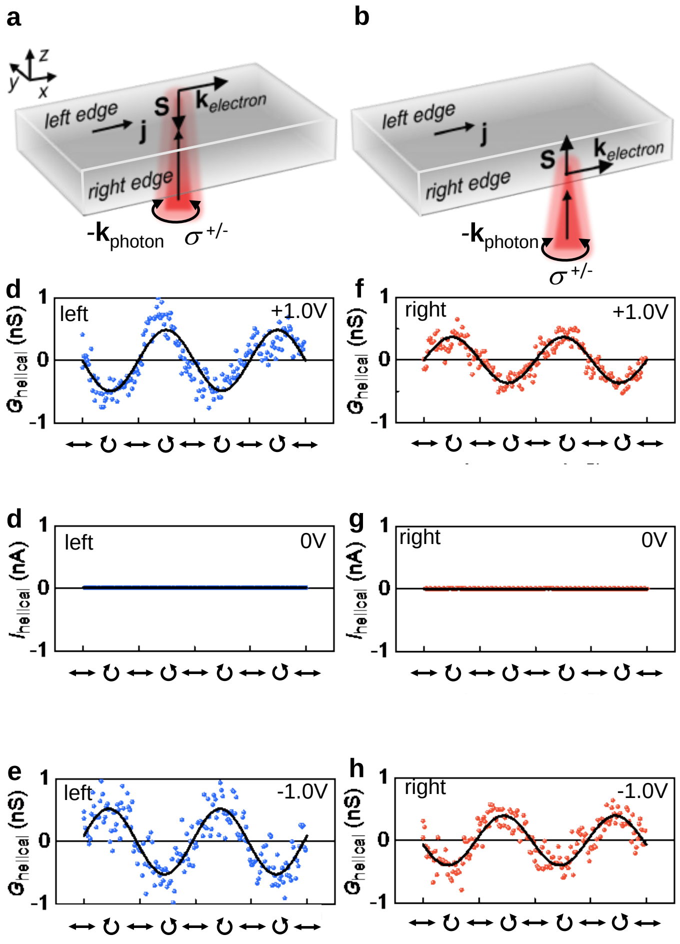

Supplementary Figure 5 | Helical photoconductance at an opposite photon wave vector. This graph shows supplementary data on the BTS platelet as discussed in Figs. 1, 3, and 4 of the main manuscript, but for a photo-excitation from the backside of the platelet. In other words, the photon wave vector is -$\mathbf{k}_{\text{photon}}$ instead of +$\mathbf{k}_{\text{photon}}$. **a,** and **b,** Schematic illustrations for the excitation configurations of the measurements in (c),(d),(e) and (f), (g), (h) respectively. **c, d, e,** Helical photoconductance G_{helical} as a function of the laser polarization at the left edge (as depicted in (a)), for applied bias voltages of V_{sd} = +1 V, V_{sd} = 0 V and V_{sd} = -1 V. **f, g, h,** Helical photoconductance G_{helical} as a function of the laser polarization at the right edge (as depicted in (b)), for applied bias voltages of V_{sd} = +1 V, V_{sd} = 0 V and V_{sd} = -1 V. We note that for V_{sd} = 0 ((d) and (g)), we plot the originally detected signal I_{helical} ~ 0 nA instead of G_{helical}, because a photoconductance cannot be defined at zero bias. The polarization is controlled with a quarter waveplate. All measurements are performed at P_{laser} = 500 μW and room temperature.

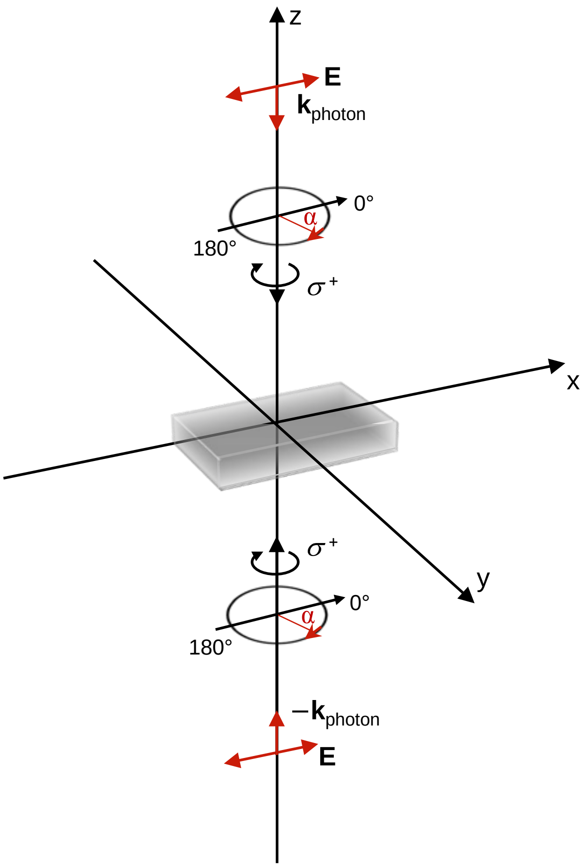

Supplementary Figure 6 | Polarization and sample configuration. Sketch of the measurement geometry and polarization optics circuitry. The quarter waveplates are arranged in a way, that the sense of rotation of the incoming laser light is defined globally and not with respect to the surface normal of the sample.

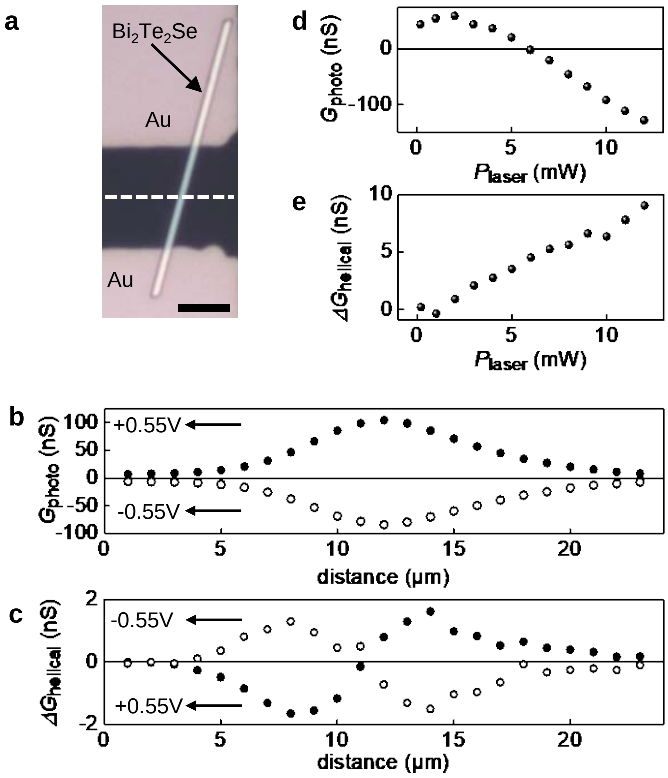

Supplementary Figure 7 | Helical photoconductance in a narrow BTS platelet. **a,** Optical microscope image of a narrow BTS nanoplatelet with a length of ~27 µm, a width of ~300 nm, and a height of 95 nm contacted by two Ti/Au contacts with a distance of 10 µm. Scale bar is 5 µm. **b,** Total photoconductance G_{photo} vs position along the white dashed line in (a) for V_{sd} = +0.55 V (full circles) and -0.55 V (open circles). The measurements are performed at room temperature and P_{laser} = 1.7 mW. **c,** Corresponding $\Delta G_{helical}$ along the white dashed line in (a). **d,** Photoconducance G_{photo} as a function of incident laser power at V_{sd} = +0.55 V. **e,** Corresponding $\Delta G_{helical}$ as a function of incident laser power at V_{sd} = +0.55 V.

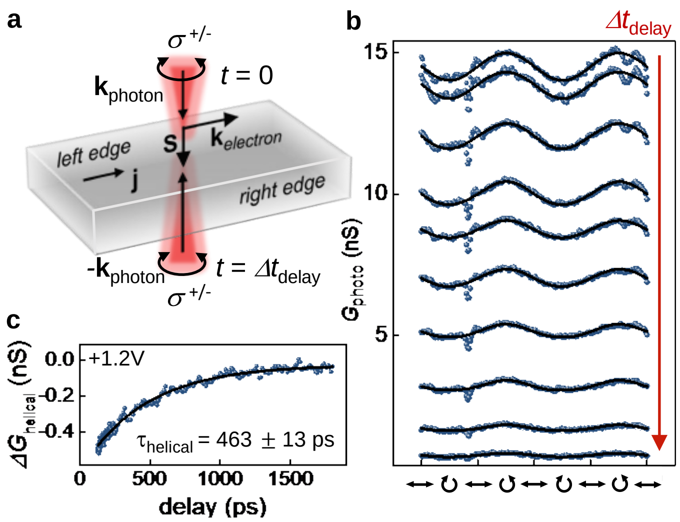

Supplementary Figure 8 | Decay time of the helical photoconductance. **a,** Schematic illustrations for measuring the auto-correlation of the helical photoconductance, when a finite bias V_{sd} is applied, and correspondingly, an electron current density **j** flows. **b,** Time-resolved pump/probe auto-correlation measurements at the left edge of a BTS platelet as a function of laser polarization. The top curve is recorded at a time-delay Δt_{delay} = 0 ps between the pump and probe pulse. The bottom curve is recorded at Δt_{delay} = 1800 ps. **c,** $\Delta G_{helical}$ as function of the time-delay Δt_{delay} between pump- and probe-pulse. Measurement parameters are P_{pump} = 1 mW, P_{probe} = 2 mW, V_{sd} = +1.2 V. The dimensions of the investigated BTS platelet are: length ~20 µm, width = 8.9 µm, height = 90 nm.

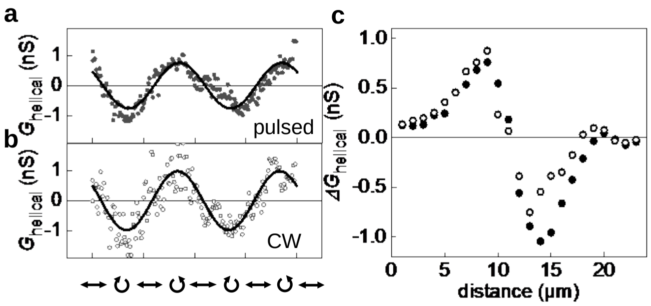

Supplementary Figure 9 | Helicity-dependent photoconductance response for pulsed and CW excitation. a, and **b,** Helicity-dependent photoconductance of the thin platelet as a function of laser polarization for a pulsed (**a,** and full circles) and CW excitation (**b,** and open circles). **c,** Corresponding $\Delta G_{helical}$ as a function of laser position. The measurements are performed at room temperature for P_{laser} = 1.7 mW and V_{sd} = -0.55V.
